# Datasets for elemental composition of 2LiF-BeF_2_ (FLiBe) salt purified by hydro-fluorination, analyzed by inductively coupled plasma mass spectrometry (ICP-MS) using two digestion methods

**DOI:** 10.1016/j.dib.2018.09.053

**Published:** 2018-10-10

**Authors:** Francesco Carotti, Bonita Goh, Martin Shafer, Raluca O. Scarlat

**Affiliations:** a University of Wisconsin-Madison, Department of Engineering Physics, USA; b University of Wisconsin-Madison & Wisconsin State Laboratory of Hygiene, Trace Element Research Group, USA

## Abstract

This article shows the elemental analysis of a batch of FLiBe prepared from LiF and BeF_2_ and purified by hydro-fluorination, see “Batch-Scale Hydrofluorination of Li_2_BeF_4_ to Support Molten Salt Reactor Development” (Kelleher et al., 2015), which was performed by the method of inductively-coupled plasma mass spectrometry (ICP-MS), with analysis samples prepared by multi-acid microwave digestion with and without HF acid. Data shows quantification of a total of sixty-five elements and is reported for a total of eight digested samples. Quantification of ^6^Li/^7^Li isotopic ratio is reported for a total of eight digested samples.

**Specifications table**

**Table:** 

Subject area	Nuclear Engineering, Chemistry, Materials Science, Chemical Engineering
More specific subject area	Molten Salt Science, Molten Fluoride Salts, Ionic Liquids
Type of data	Column graph and table
How data was acquired	Inductively coupled plasma mass spectrometry (ICP-MS)
Data format	Raw, analyzed
Experimental factors	A 1 kg batch of FLiBe was transferred in liquid form to a Ni vessel, from a 57 kg batch that was purified by hydrofluorination by Kelleher et al. [1], then poured and frozen nickel trays. All handling and storage was performed under high-purity Argon gas. Two solid samples of FLiBe were ground and then dissolved in acidic aqueous solutions by microwave-aided digestion. Each digest was analyzed in triplicate on the ICP-MS.
Experimental features	Quantitative elemental analysis of purified 2LiF-BeF_2_ (FLiBe)
Data source location	Madison, Wisconsin, USA
Data accessibility	Data is included in this article
Related research article	*n/a*

**Value of the data**

• Data can be used as a baseline for elemental composition of purified FLiBe, relevant to groups studying chemical or thermophysical properties of FLiBe
• Data can be used as partial evidence indicating the effectiveness of hydro-fluorination as a purification step of FLiBe.
• Data presented here can be relevant to groups investigating aqueous digestion methods for FLiBe.
• Data presented here can be relevant to design of nuclear reactors that use FLiBe as a coolant or fuel solvent.
• Data presented here can serve as a benchmark for ICP-MS measurement sensitivity for the set of elements that were analyzed here.

## 1 Data

The ICP-MS data is reported in Table 1. The assumed isotopic abundance for each element analyzed is reported in Table 2. Each sample was independently digested in duplicate. Each solution was analyzed in triplicate. The values reported here are the average and standard deviation of the three ICP-MS analyses. The date of FLiBe sample collection was 2018-04-09; the date of sample digest was 2018-04-11, the date of ICP-MS analysis was 2018-04-18 (analytical batch B1714 at the Wisconsin State Laboratory of Hygiene).

**Table 1:** ICP-MS Data for hydrofluorinated 2LiF-BeF2 handled in nickel containers under Argon atmosphere. Two solid samples: Sample 1 and Sample 2. Two powder samples for each solid sample (e.g. Sample 1 and Sample 1 Duplicate). Elements reported in order of abundance. Standard deviation among multiple ICP-MS runs on the same digest solution. (See supplementary material).

(a) The isotope analyzed by masss pectroscopy is indicated in this table. The μg/g values refer to the element, assuming natural isotopic abundance for all elements (including Li), as reported in Table 2.(b) Dilutions of 1:2,000 and 1:10,000 were analyzed for Li and Be; 1:1,200 for 6Li/7Li ratio; 1:5 for all other elements.

Natural isotopic abundance is assumed for all elements.2017-06-07. Francesco Carotti, Bonita Goh, Martin Shafer, Raluca O. Scarlat. roscarlat@wisc.edu

**Table 2:** Isotope natural abundance values used in the interpretation of the ICP-MS data. (See supplementary material).

2017-06-01. Martin Shafer, Raluca O. Scarlat. roscarlat@wisc.edu

## 2 Experimental design, materials and methods

### 2.1 FLiBe preparation

A 1 kg batch of FLiBe was transferred in liquid form to a nickel vessel (Ni 200 alloy), from a 57 kg FLiBe batch purified by hydrofluorination and reduced with hydrogen sparging. The detailed purification procedure was reported by Kelleher et al. [1]. The 1 kg batch was subsequently poured and frozen in small (10 cm x 10 cm) nickel trays (Ni 200 alloy). The frozen salt was then removed from the nickel trays, and broken up into small pieces, for sampling. Two such solid 0.5 g samples (Sample 1 and Sample 2) were collected and sent for ICP-MS analysis. Handling and storage in all the steps was performed under ultra-high purity argon gas (UHP, Airgas).

### 2.2 FLiBe digestion for ICP-MS

The solid salt sample of ~0.5 g was ground using a small agate mortar and pestle within a fume-hood, with the goal of relatively uniform particle size; very fine powdering is not necessary for the microwave digestion. The duration of the grinding process is on the order of 10 min. Samples of < 100 mg were taken from this powder for digestion; the weight of each digested powder sample is reported in Table 1

Each powder sample of FLiBe was dissolved in 2.00 mL of Millipore high-purity water and digested with one of the following two acidic solutions:

• Acidic solution with HF: 7.00 mL 16 M Nitric Acid, 3.00 mL 12 M Hydrochloric Acid, 2.00 mL 28 M Hydrofluoric Acid and 3.00 mL MQ.
• Acidic solution without HF: 8.00 mL 16 M Nitric Acid, 4.00 mL 12 M Hydrochloric Acid, and 3.00 mL MQ.

This digestion was topped up to 50.00 mL with water. Microwave-aided Teflon-bomb digestion was performed using Milestone Ethos EZ instrument with SK12 and PRO24 microwave rotors. Details on the methodology can be found in [2] and [3]. Digested samples were further diluted 1:5, 1:1,200, 1:2,000 and 1:10,000 to achieve optimum ion count rate for quantification on the ICP-MS instrument.

Samples for each ICP-MS batch comprised of: NIST SRM Marine Sediment [4] and San Joaquin Soil [5], aqueous mixed-metal digestion bomb spikes [6], bottle blanks, sample spikes [6], sample duplicates and reagent (method) blanks. In the case of aqueous mixed metal digestion bomb spikes, mixed element spike solutions were used to verify digestion recovery and these were prepared from NIST traceable single element standards purchased from High Purity Standards Inc [6]. In the case of sample spikes, mixed element spike solutions were used to verify element recovery in paired digested samples, and these were also prepared from NIST traceable single element standards purchased from High Purity Standards Inc. Quality assurance (QA) samples included initial calibration blanks (ICB), initial calibration verification (ICV), continuing calibration blank (CCB), continuing calibration verification (CCV). Each analytical batch contained 50% QA samples and 50% analysis samples. Mixed-element calibration verification solutions (ICV) were prepared from a second source vendor of NIST-traceable single-element standards [7]. CCV samples were prepared from NIST traceable single element solution standards purchased from High Purity Standards Inc [6]. Each element analyzed was included in the spikes, and three target spiking levels were used: 0.2 µg/L for Au, Ce, Cs, Dy, Er, Eu, Gd, Hf, Ho, Ir, La, Lu, Nb, Nd, Pr, Pt, Re, Rh, Ru, Sm, Ta, Tb, Th, Tm, Y, Yb, Zr; 400 µg/L for Al, Be, Ca, Fe, K, Li, Mg, Na, P, S; and 2.0 µg/L for all other elements.

### 2.3 ICP-MS analysis

Samples were run on a Thermo-Fisher Scientific Element 2 instrument (formerly Thermo-Finnigan). To eliminate spectral interferences, ICP-MS instrument settings were adjusted among low resolution (LR), medium resolution (MR), and high resolution (HR). The instrument setting used, and the isotope analyzed for each element are indicated in Table 1. Each solution was analyzed in triplicate on the ICP-MS, for statistics.

The ICP-MS measures ion counts per second. Each specific isotope has slightly different transmission characteristics in the mass spectrometer (i.e. sensitivity), therefore a mass bias correction is applied. This bias also changes slightly during a run due to cone aging and other effects. The absolute bias and relative bias are corrected by a "standard-sample-standard-sample" approach: known standards are run after each sample; the measured ratios in these standards are used to correct the bias in the samples. The standard deviation for the concentration values is based on three measured values, from three ion count data blocks. Each ion count data block is typically based on 30 ion counts. For potassium, acid-matrix matched calibration checks were used, using relevant potassium levels at frequent locations in the analytical sequence.

For isotope ratio measurements, the mass spectrometer is run in a specific mode: the magnet is fixed on a mass between the two isotopes of interest and the electrostatic analyzer is rapidly scanned between those isotopes and data for 10 scans are collected. The ion counts at each isotope in each scan are baseline (blank) corrected and then isotope ratio is computed. The average of these 10 ratios is then calculated. This is called a single block of data. This whole process is repeated 9 more times to generate 10 blocks of data, each with an average value. The average (and standard deviation or standard error) of the 10 averages is what is used to generate the final isotope ratio value.

The Li standard used to compare isotopic ratios was procured from High Purity Standards (HPS), product number 100029-1 (1000 µg/mL Li in 1% HNO_3_) and is directly traceable to NIST SRM 3129. We note that the HPS and NIST standards are certified for concentration, but they are not certified for isotope ratios. The NIST standard that is certified for Li isotope ratios (reference material RM8545-LSVEC) has been unavailable for more than a year; it has a true absolute abundance of 0.08215 (mol/mol), identical to the value we measured for the HPS standard, which was 0.0821 ± 0.0002 (mol/mol).

## Footnotes

Appendix A Supplementary data associated with this article can be found in the online version at https://doi.org/10.1016/j.dib.2018.09.053.
