# Supplementary material for: Datasets for elemental composition of 2LiF-BeF2 (FLiBe) salt purified by hydro-fluorination, analyzed by inductively coupled plasma mass spectrometry (ICP-MS) using two digestion methods
Source: Data Brief. 2018 Oct 10;21:1612–7. doi: 10.1016/j.dib.2018.09.053 (PMC6249393; doi:10.1016/j.dib.2018.09.053)
Supplement: Supplementary file 1 — Supplementary material [file mmc1.pdf]

DIB-D-18-01737
Declarations of Interest: None

Sept. 18, 2018

I do not have any financial or personal relationships with other people or organizations that could inappropriately influence (bias) the work reported in this manuscript.

Raluca Scarlat
